# The Association Between Depression and Survival in Asthma: The Mediating Role of Income

**DOI:** 10.1155/da/8153352

**Published:** 2026-09-19

**Authors:** Zhiyi Wang, Xiaojing Teng

**Affiliations:** ^1^ Department of Clinical Laboratory, Hangzhou Women’s Hospital (Hangzhou Maternity and Child Health Care Hospital), Hangzhou, Zhejiang, China, womanhospital.cn; ^2^ Department of Medical Clinical Laboratory, Affiliated Hangzhou First People’s Hospital, School of Medicine, Westlake University, Hangzhou, Zhejiang, China, westlake.edu.cn

**Keywords:** asthma, depression, life expectancy, mediation analysis, mortality

## Abstract

**Background:**

Depression is common among individuals with asthma and is linked to increased mortality. However, whether socioeconomic factors contribute to this association remains unclear. We explored whether income may partially account for the association between depressive symptoms and reduced survival in adults with asthma.

**Methods:**

A total of 4945 asthma patients were included using baseline data from the National Health and Nutrition Examination Survey (NHANES) 2005–2018 linked to the National Death Index (NDI) mortality follow‐up through December 31, 2019. Depression was assessed using the Patient Health Questionnaire‐9 (PHQ‐9), and mortality was identified through linkage to the NDI. Cox proportional hazards models were used to evaluate the association between depression symptoms and all‐cause mortality, and restricted mean survival time (RMST) analysis quantified survival differences over 15 years. Exploratory mediation analysis with quasi‐Bayesian simulation was conducted to evaluate whether the poverty‐income ratio (PIR) may partially account for the observed association, adjusting for demographic, behavioral, and clinical covariates.

**Results:**

Depression was associated with all‐cause mortality in asthma patients, and the strength of this association decreased after adjustment for demographic, behavioral, and clinical factors. Over a 15‐year period, patients with depression symptoms (PHQ‐9 ≥ 10) had markedly shorter survival, losing 9.89 months in RMST (155.2 vs. 165.1 months; *p* < 0.001). In exploratory mediation analysis, PIR accounted for 23.8% of the observed association between depressive symptoms and reduced survival, suggesting a potential socioeconomic contribution to the survival gap.

**Conclusion:**

Depressive symptoms were associated with reduced survival among adults with asthma, and income may partially account for this observed relationship. These findings should be interpreted cautiously because depressive symptoms and income were measured at baseline, limiting causal interpretation of the mediation analysis. Further longitudinal studies are needed to confirm these findings and clarify temporal relationships.

## 1. Introduction

Asthma is a chronic respiratory condition that affects millions of individuals worldwide, with significant implications for both physical health and quality of life. Recent studies have highlighted the substantial burden of comorbid mental health conditions [1], particularly depression, in asthma patients [2, 3]. Depression is associated with increased morbidity [4], impaired treatment adherence, and poorer health outcomes in asthma populations [5, 6]. At the same time, the economic impact of asthma and mental health disorders cannot be ignored [7] as lower income levels may exacerbate both the physical and psychological burden of asthma, further complicating the management of the disease.

Despite growing awareness of the relationship between asthma and mental health, the mechanisms underlying these associations remain poorly understood. Specifically, it is unclear how income, an important social determinant of health, may partially account for the observed association between depression and mortality in individuals with asthma [8]. In the present study, income was measured using the poverty–income ratio (PIR), a U.S.‐specific measure that reflects household income relative to the federal poverty threshold. Therefore, low‐ and high‐income categories in this analysis should be interpreted within the socioeconomic and healthcare context of the United States rather than as directly comparable income categories across countries. In particular, low income in the United States may not correspond to low income in low‐ and middle‐income countries, where poverty thresholds, healthcare financing, social welfare systems, and access to care may differ substantially. While prior studies have recognized income as a confounder, its potential explanatory role in the association between depression and mortality remains underexplored. Given the rising prevalence of both asthma and depression, as well as the growing disparities in income levels among different population groups [9], it is essential to investigate how socioeconomic factors may contribute to the exacerbation of mental health and mortality outcomes in asthma patients [10].

This study aims to address this research gap by examining the association between depression and all‐cause mortality among asthma patients. Utilizing data from the National Health and Nutrition Examination Survey (NHANES) from 2005 to 2018 and linked mortality data from the National Death Index (NDI), we conducted a retrospective cohort analysis to explore whether household income, measured by PIR, may partially account for the observed association between depression and survival outcomes in this U.S. population. In the U.S. healthcare context, depression may influence asthma outcomes not only through psychological distress but also through reduced treatment adherence, impaired self‐management, limited healthcare access, and financial barriers to care. Structural inequalities related to income, insurance coverage, and access to regular care may therefore shape the relationship among depression, income, and mortality. Our findings may provide population‐based evidence from the United States on socioeconomic mechanisms related to health disparities among individuals with asthma and inform public health interventions aimed at improving health outcomes in countries or settings with comparable socioeconomic and healthcare structures.

## 2. Methods

### 2.1. Study Design and Data Sources

This retrospective cohort study utilized baseline data from the NHANES 2005–2018, which were collected in 2‐year cycles as part of a nationwide health and nutrition survey conducted in the United States [11]. Mortality outcomes were ascertained through linkage to the NDI up to December 31, 2019 [12]. Baseline exposure, income, and covariate information were obtained from NHANES interviews, examinations, and laboratory data, whereas mortality status and follow‐up time were obtained from the NHANES‐linked mortality file. Self‐reported physician‐diagnosed asthma was identified using the NHANES variable MCQ010 (“Has a doctor ever told you that you have asthma?”). Participants aged ≥18 years with self‐reported physician‐diagnosed asthma were eligible for inclusion.

**Figure 1:**
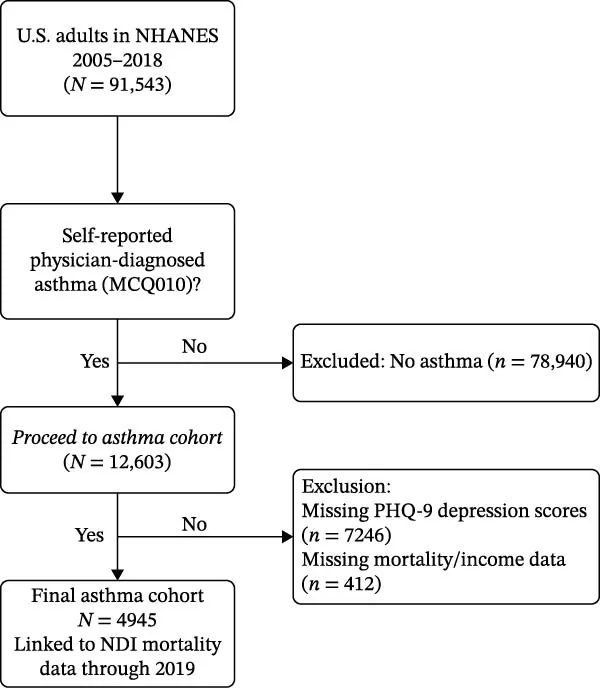
Flowchart of participant selection from NHANES 2005–2018. This flowchart outlines the selection process for the final asthma cohort. From the initial NHANES sample of 91,543 U.S. adults, participants were first screened for self‐reported physician‐diagnosed asthma. Those without asthma (*n* = 78,940) were excluded. The remaining 12,603 individuals who reported asthma were further excluded for missing depression scores (PHQ‐9) and missing mortality or income data, resulting in a final asthma cohort of 4945 individuals linked to mortality data through the National Death Index (NDI) through 2019.

From an initial sample of 91,543 participants, we excluded individuals without asthma (*n* = 78,940), missing depression scores (*n* = 7246), or incomplete mortality/income data (*n* = 412), yielding a final analytic cohort of 4945 adults with asthma (Figure 1).

NHANES protocols were approved by the National Center for Health Statistics Research Ethics Review Board, and all participants provided written informed consent. The present study used publicly available deidentified NHANES data and involved secondary analysis of existing data; therefore, additional informed consent from participants was not required.

### 2.2. Assessment of Depression

Depression was assessed using the 9‐item Patient Health Questionnaire (PHQ‐9), a validated screening tool for depressive symptoms. The PHQ‐9 evaluates symptoms over the preceding 2 weeks, with total scores ranging from 0 to 27 [13]. The categorization of depression severity followed the commonly used PHQ‐9 cutoffs established by Kroenke et al. [14], with participants classified as follows [15]: nondepression, PHQ‐9 < 10; depression, PHQ‐9 ≥ 10; and severe depression, PHQ‐9 > 18. PHQ‐9 ≥ 10 was used as the primary binary definition of moderate‐to‐severe depressive symptoms, while PHQ‐9 >18 was used only in subgroup analysis to examine severe depressive symptoms. The PHQ‐9 score was also analyzed as a continuous variable to evaluate the association per unit increase in depressive symptom burden. The NHANES variable PHQSCORE was used to extract these data. Because PHQ‐9 is a screening instrument, PHQ‐9 ≥ 10 was interpreted as moderate‐to‐severe depressive symptoms rather than a clinical diagnosis of major depressive disorder.

### 2.3. Assessment of Income

Income was operationalized using the PIR, calculated as the ratio of family income to the federal poverty threshold, as defined by the U.S. Department of Health and Human Services (DHHS) guidelines. Because PIR is defined relative to the U.S. federal poverty threshold, the income categories in this study should be interpreted within the socioeconomic and healthcare context of the United States rather than as directly comparable income categories across countries. NHANES is designed to represent the noninstitutionalized U.S. population; therefore, the socioeconomic context of this study reflects a U.S. population‐based sampling framework rather than a specific local or regional geographic setting. Participants were stratified into the following income categories: low income PIR < 1.30, corresponding to household income below 130% of the federal poverty level; middle income, PIR 1.30–3.49; and high income, PIR ≥ 3.50. This categorization follows the commonly used thresholds for income stratification, which are based on the federal poverty guidelines [16]. The PIR variable was used as a covariate in the adjusted models and was further examined as a potential explanatory factor in the exploratory mediation analysis.

### 2.4. Outcome and Follow‐Up

The primary outcome was all‐cause mortality. Mortality status was obtained from the NHANES Linked Mortality File, which links eligible NHANES participants to the NDI. The mortality file was merged with the analytic NHANES dataset using the unique participant identifier (SEQN). Mortality status was defined using MORTSTAT (1 = deceased and 0 = alive), and mortality follow‐up was available through December 31, 2019. The follow‐up duration was calculated in months using PERMTH_INT, representing the time from the NHANES interview date to death or censoring on December 31, 2019.

### 2.5. Data Collection

Data were collected through structured interviews, physical examinations, and laboratory tests following standardized NHANES protocols. Demographic variables included age (continuous), sex (male/female), and race/ethnicity (Mexican American/other Hispanic/non‐Hispanic White/non‐Hispanic Black/other race). Socioeconomic factors encompassed education level (categorized as <high school, high school, or some college/higher), marital status (married, widowed, divorced, separated, never married, or living with partner), and (PIR; <1.30, 1.30–3.49, or ≥3.50). Behavioral factors included smoking status (current smoker: yes/no), while clinical measures comprised body mass index (BMI: <18.5, 18.5–24.9, or ≥25.0 kg/m^2^), diabetes (self‐reported diagnosis), and hypertension (self‐reported diagnosis).

### 2.6. Statistical Analysis

All analyses were conducted using R software (Version 4.2.2) with the rms and survival packages, and EmpowerStats (Version 4.2). NHANES complex survey weights (WTSA2YR) were applied to account for the stratified multistage sampling design. Continuous variables were presented as survey‐weighted means with 95% confidence intervals (CIs), and categorical variables were presented as survey‐weighted percentages with 95% CIs. Group differences were assessed using survey‐weighted linear regression for continuous variables and survey‐weighted chi‐square tests for categorical variables. Survival analyses were conducted using mortality status and follow‐up time obtained from the NHANES Linked Mortality File. In this retrospective cohort design, time was defined as the number of months from the NHANES interview date to death or censoring using the variable PERMTH_INT. The start point of follow‐up was the NHANES interview date, and the end point was the date of death or censoring on December 31, 2019, whichever occurred first. Mortality status was incorporated using MORTSTAT, with participants classified as deceased or alive/censored. Cox proportional hazards models estimated hazard ratios (HRs) and 95% CIs for the depression‐mortality association, with sequential adjustments: no adjust, Adjust I (gender, age, race/ethnicity), and Adjust II (education level, smoking, diabetes, PIR, marital status, BMI, and hypertension awareness).

In addition, to evaluate the independent association between income and mortality, an additional Cox regression analysis was performed using PIR as the main independent variable, with all‐cause mortality as the outcome. PIR was analyzed as a continuous variable and as a categorical variable, with PIR ≥ 3.50 used as the reference group.

To evaluate whether the depression–mortality association differed by asthma status, an additional Cox regression analysis was conducted among participants with and without asthma by including an interaction term between depressive symptoms and asthma status. Stratified estimates were further presented for participants with asthma and those without asthma.

Restricted mean survival time (RMST) analysis was performed to compare unadjusted absolute survival differences between depression groups at a predefined time horizon (*τ* = 180 months), addressing nonproportional hazards. The 15‐year horizon was selected because it provides a clinically interpretable long‐term survival window in NHANES‐linked mortality data while limiting instability from sparse follow‐up at the tail of the survival curve. RMST was interpreted as a support unadjusted measure of absolute survival difference and was considered alongside the multivariable Cox regression models. Kaplan–Meier survival curves were generated to visualize unadjusted survival probabilities over time, with statistical significance assessed using weighted log‐rank tests.

Exploratory mediation analysis employed the Sobel test and quasi‐Bayesian simulations (10,000 iterations) to estimate the indirect effect involving income in the observed association between depressive symptoms and all‐cause mortality. In the primary Cox regression models, PIR was included as a covariate to adjust for socioeconomic confounding, whereas in the exploratory mediation analysis, PIR was examined as a potential explanatory factor in a hypothesized pathway. This analysis assumes no unmeasured confounding of the exposure–outcome, exposure–mediator, and mediator–outcome relationships. Because depressive symptoms, PIR, and covariates were measured at baseline, temporal ordering could not be definitively established. Therefore, the estimated mediation proportion was interpreted as exploratory and assumption‐dependent rather than as definitive causal evidence. Missing covariate data were handled using dummy variables. Statistical significance was defined as *p* < 0.05.

## 3. Results

### 3.1. Asthma Patient Characteristics by Depression Status

The study included 4945 adults with asthma, stratified into nondepression (PHQ‐9 < 10, *n* = 4247) and depression (PHQ‐9 ≥ 10, *n* = 698) groups. Participants with depression were significantly older than those without depression, with a survey‐weighted mean age of 45.60 years (95% CI: 44.08–47.11) versus 43.71 years (95% CI: 42.95–44.48; *p* = 0.0243). There was a statistically significant difference in race/ethnicity distribution between groups (*p* = 0.0124), with depressed patients having a higher proportion of other Hispanic participants (7.72% vs. 4.84%). Socioeconomic disparities were evident: depressed patients exhibited higher proportions with less than high school education (26.07% vs. 11.74%), lower income (PIR < 1.30: 47.98% vs. 23.50%), and higher rates of divorce (19.48% vs. 10.08%, all *p* < 0.001). Behavioral and clinical profiles differed significantly between the groups. Current smoking was significantly more prevalent among depressed individuals (69.50% vs. 44.26%, *p* < 0.001), and they also reported higher rates of diabetes (19.37% vs. 9.77%, *p* < 0.001) and hypertension awareness (49.88% vs. 32.97%, *p* < 0.001). BMI distributions did not significantly differ between groups (*p* = 0.231), with overweight or obesity (BMI ≥ 25.0) common in both cohorts (75.81% vs. 70.88%). Mortality was significantly higher among depressed participants (11.68% vs. 6.40%, *p* < 0.001) (Table 1).

**Table 1:** Baseline characteristics of asthma patients by depression status.

Characteristics	Nondepression	Depression	*p*‐Value
	PHQ‐9 < 10 *N* = 4247	PHQ‐9 ≥ 10 *N* = 698	
Age (years)	43.71 (42.95, 44.48)	45.60 (44.08, 47.11)	0.0243
Gender (%)	—	—	0.0014
Male	42.67 (40.52, 44.85)	33.44 (28.83, 38.39)	—
Female	57.33 (55.15, 59.48)	66.56 (61.61, 71.17)	—
Race/ethnicity (%)	—	—	0.0124
Mexican American	5.30 (4.42, 6.33)	4.42 (2.98, 6.53)	—
Other Hispanic	4.84 (3.94, 5.92)	7.72 (5.76, 10.27)	—
Non‐Hispanic White	70.38 (67.50, 73.10)	66.83 (61.83, 71.47)	—
Non‐Hispanic Black	12.67 (11.00, 14.55)	13.67 (11.24, 16.53)	—
Other race	6.82 (5.77, 8.04)	7.36(5.53,9.73)	—
Education level (%)	—	—	<0.0001
Less than high school	11.74 (10.23, 13.45)	26.07 (21.94, 30.67)	—
High School	20.66 (18.87, 22.57)	25.05 (21.23, 29.30)	—
Some college or higher	62.62 (60.29, 64.89)	46.80 (41.71, 51.95)	—
Missing	4.98 (4.27, 5.79)	2.08 (1.21, 3.57)	—
Marital status (%)	—	—	<0.0001
Married	49.85 (47.72, 51.98)	34.75 (29.94, 39.90)	—
Widowed	4.99 (4.24, 5.87)	5.91 (4.29, 8.10)	—
Divorced	10.08 (9.03, 11.23)	19.48 (15.75, 23.85)	—
Separated	2.24 (1.78, 2.82)	7.28 (5.17, 10.16)	—
Never married	21.20 (19.48, 23.04)	18.80 (15.05, 23.23)	—
Living with partner	7.31 (6.34, 8.42)	11.62 (8.78, 15.22)	—
Smoking (%)	—	—	<0.0001
Yes	44.26 (42.08, 46.47)	69.50 (64.62, 73.98)	—
No	52.97 (50.80, 55.13)	29.34 (24.86, 34.27)	—
Missing	2.77 (2.30, 3.32)	1.15 (0.54, 2.45)	—
Diabetes (%)	—	—	<0.0001
Yes	9.77 (8.61, 11.07)	19.37 (16.04, 23.20)	—
No	88.45 (87.08, 89.69)	75.63 (71.21, 79.56)	—
Missing	1.78 (1.42, 2.23)	5.00 (2.65, 9.25)	—
Hypertension awareness (%)	—	—	<0.0001
Yes	32.97 (30.93, 35.08)	49.88 (44.34, 55.43)	—
No	67.03 (64.92, 69.07)	49.99 (44.43, 55.54)	—
Missing	0.00 (0.00, 0.00)	0.13 (0.02, 0.91)	—
PIR (%)	—	—	<0.0001
<1.30	23.50 (21.60, 25.51)	47.98 (42.92, 53.07)	—
1.30–3.49	34.18 (31.85, 36.59)	33.35 (28.45, 38.63)	—
≥3.50	42.32 (39.37, 45.33)	18.68 (14.44, 23.81)	—
BMI (%)	—	—	0.2305
<18.5	1.67 (1.24, 2.26)	2.14 (0.65, 6.80)	—
18.5–24.9	26.78 (24.88, 28.77)	20.99 (16.94, 25.71)	—
≥25.0	70.88 (68.83, 72.85)	75.81 (70.51, 80.42)	—
Missing	0.67 (0.44, 1.01)	1.06 (0.46, 2.43)	—
MORTSTAT (%)	—	—	<0.0001
Live	93.60 (92.52, 94.53	88.32 (85.48, 90.66)	—
Die	6.40 (5.47, 7.48)	11.68 (9.34, 14.52)	—

*Note:* Continuous variables are presented as survey‐weighted means with 95% confidence intervals (CIs), and categorical variables are presented as survey‐weighted percentages with 95% CIs. Group differences were assessed using survey‐weighted linear regression for continuous variables and survey‐weighted chi‐square tests for categorical variables. NHANES survey weights were applied. Statistical significance was set at *p*  < 0.05. Missing data: education level 383 (7.75%), smoking 236 (4.77%), marital status 301 (6.09%), BMI 53 (1.07%), hypertension awareness 1 (0.02%), diabetes 117 (2.37%).

Abbreviation: BMI, body mass index.

### 3.2. Depression Symptoms and Their Association With All‐Cause Mortality in Asthma Patients

In Cox proportional hazards models, the PHQ‐9 score was associated with all‐cause mortality among adults with asthma. In the fully adjusted model, each one‐point increase in PHQ‐9 score was associated with a modestly higher mortality risk (HR = 1.04, 95% CI: 1.02–1.06, *p* < 0.0001). When PHQ‐9 was analyzed categorically, participants with PHQ‐9 scores of 10–18 had a higher mortality risk than those with PHQ‐9 scores ≤9 after full adjustment (HR = 1.41, 95% CI: 1.10–1.82, *p* = 0.0072). However, the association for PHQ‐9 > 18 was attenuated and no longer statistically significant after full adjustment (HR = 1.23, 95% CI: 0.72–2.08, *p* = 0.4486), suggesting that the findings should be interpreted cautiously (Table 2).

**Table 2:** Adjusted hazard ratios for all‐cause mortality by depression severity in asthma patients.

Variable	No adjust		Adjust I		Adjust II	
	HR (95% CI)	*p*‐Values	HR (95% CI)	*p*‐Values	HR (95% CI)	*p*‐Values
PHQSCORE	1.05 (1.03, 1.06)	<0.0001	1.07 (1.05, 1.08)	<0.0001	1.04 (1.02, 1.06)	<0.0001
PHQSCORE categorical						
≤9	Ref	—	Ref	—	Ref	—
10–18	1.83 (1.45, 2.32)	<0.0001	2.17 (1.71, 2.76)	<0.0001	1.41 (1.10, 1.82)	0.0072
>18	1.40 (0.83, 2.34)	0.2033	1.88 (1.12, 3.16)	0.0178	1.23 (0.72, 2.08)	0.4486
*p* for trend	<0.0001		<0.0001		0.0212	

*Note:* Cox proportional hazards regression models were used to examine the association between PHQSCORE and all‐cause mortality. Results are presented as hazard ratios (HRs) with 95% confidence intervals (CIs) and corresponding *p*‐values. PHQSCORE was analyzed as a continuous variable, a continuous categorical variable, and a categorical variable (≤9 as the reference group). Adjust I model adjusted for gender, age, race/ethnicity. Adjust II model additionally adjusted for education level, smoking, diabetes, PIR, marital status, BMI, hypertension awareness. The time variable in the Cox models was PERMTH_INT (time to event in months). Statistical significance was defined as *p* < 0.05.

In additional Cox regression analyses treating PIR as the main independent variable, higher PIR was associated with a lower all‐cause mortality risk. In the fully adjusted model, continuous PIR was inversely associated with mortality risk (HR = 0.80, 95% CI: 0.74–0.87, *p* < 0.0001). Compared with participants with high income (PIR ≥ 3.50), those with middle income (PIR 1.30–3.49) and low income (PIR < 1.30) had higher mortality risks, with HRs of 1.72 (95% CI: 1.26–2.35, *p* = 0.0007) and 2.23 (95% CI: 1.60–3.11, *p* < 0.0001), respectively (Table S1).

In an additional analysis including both asthmatic and nonasthmatic participants, the interaction between depressive symptoms and asthma status was not statistically significant in the fully adjusted model (*p* for interaction = 0.7033), suggesting that the depression–mortality association did not differ significantly by asthma status. The stratified results are presented in Table S2.

### 3.3. Exploratory Mediation Analysis of PIR in the Depression–Mortality Association

**Figure 2:**
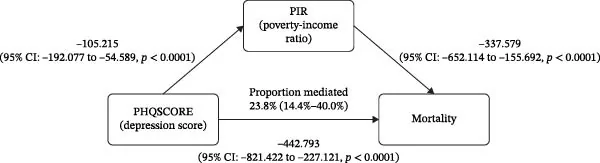
Exploratory mediation analysis of poverty‐income ratio (PIR) in the observed association between depressive symptoms and mortality. This figure illustrates the exploratory mediation framework involving depressive symptoms (PHQ‐9 ≥ 10), poverty‐income ratio (PIR), and all‐cause mortality. The total observed association between depressive symptoms and mortality was estimated as −442.793 days (95% CI: −821.422 to −227.121, *p* < 0.0001). The exploratory analysis suggested that PIR accounted for 23.8% (95% CI: 14.4%–40.0%) of this observed association. The indirect component involving PIR was −105.215 days (95% CI: −192.077 to −54.589, *p* < 0.0001), and the direct component not accounted for by PIR was −337.579 days (95% CI: −652.114 to −155.692, *p* < 0.0001).

Exploratory mediation analysis showed that income (PIR) accounted for 23.8% (95% CI: 14.4%–40.0%; *p* < 0.001) of the total observed association between depressive symptoms (PHQ‐9 ≥ 10) and mortality among asthma patients. PHQ‐9 ≥ 10 was associated with an estimated 442.8‐day reduction in survival (95% CI: −821.4 to −227.1; *p* < 0.001), with a direct component not accounted for by income of −337.6 days (95% CI: −652.1 to −155.7; *p* < 0.001) and an indirect component involving income of −105.2 days (95% CI: −192.1 to −54.6; *p* < 0.001) (Table 3, Figure 2). These findings suggest that income disparities may partially account for the observed association between depressive symptoms and mortality among asthma patients. However, because depressive symptoms and PIR were measured at baseline, this mediation proportion should be interpreted as exploratory and assumption‐dependent rather than as definitive causal evidence.

**Table 3:** Results of exploratory mediation analysis examining PIR in the observed association between depressive symptoms and mortality in asthma patients.

Effect type	Estimate	95% CI lower	95% CI upper	*p*‐Value
Total effect	−442.793	−821.422	−227.121	<0.0001
Mediation effect	−105.215	−192.077	−54.589	<0.0001
Direct effect	−337.579	−652.114	−155.692	<0.0001
Proportion mediated (%)	23.8	14.4	40.0	<0.0001

*Note:* Depression (PHQ‐9 ≥ 10) was the exposure, with income (PIR) as mediator. Adjusted for demographics, education, smoking, marital status, hypertension, BMI, and diabetes. Mediation effects estimated using quasi‐Bayesian Monte Carlo (10,000 iterations) with bootstrap CIs. Mortality modeled via Cox regression; effects shown as survival days (negative = shorter survival).

### 3.4. Survival Disparities by Depression Status

**Figure 3:**
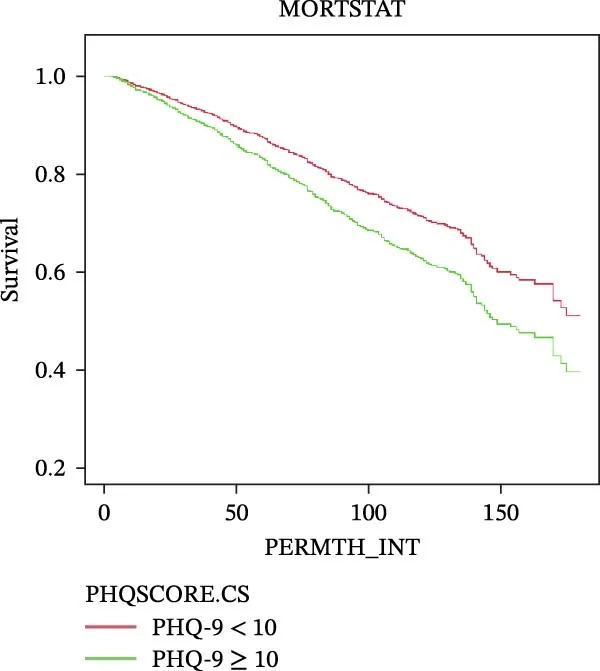
Kaplan–Meier survival curves by depression status in asthma patients. Kaplan–Meier survival curves compare unadjusted survival probabilities between asthma patients with depressive symptoms (PHQ‐9 ≥ 10) and those without depressive symptoms (PHQ‐9 < 10). The curves show lower unadjusted survival probability over time among participants with depressive symptoms.

Kaplan–Meier curves revealed significantly lower survival probabilities among asthma patients with depression symptoms (PHQ‐9 ≥ 10) compared to those without depression (Figure 3). Survival differences emerged early within the first 2 years and widened progressively over the follow‐up period. RMST analysis indicated that participants with depressive symptoms had nearly 10 months shorter unadjusted RMST over a 15‐year period (tau = 180 months): RMST was 155.2 ± 2.24 months (95% CI: 150.8–159.6) for participants with depressive symptoms versus 165.1 ± 0.73 months (95% CI: 163.7–166.6) for participants without depressive symptoms (Table 4). Correspondingly, participants with depressive symptoms experienced an average of 24.8 months lost (RMTL) compared to 14.9 months in nondepressed counterparts.

**Table 4:** Restricted mean survival time by depression status in asthma patients: A 15‐year RMST analysis.

Group	RMST (months)	SE	95% CI	RMTL (months)	SE	95% CI
Depression (PHQ‐9 ≥ 10)	155.239	2.242	150.845–159.634	24.761	2.242	20.366–29.155
Nondepression (PHQ‐9 < 10)	165.133	0.725	163.713–166.554	14.867	0.725	13.446–16.287

*Note*: Restricted mean survival time (RMST) and restricted mean time lost (RMTL) were calculated over a 15‐year follow‐up period (tau = 180 months). Group comparisons were derived from unadjusted RMST analysis. RMST: average survival time within the restricted period; RMTL: average time lost due to mortality within the restricted period; PHQ‐9 ≥ 10: classified as depression; PHQ‐9 < 10: nondepression reference group. RMST difference: 9.9 months (depression vs. nondepression).

Abbreviations: CI: confidence interval; SE, standard error.

These results align with Cox regression findings (Table 2), suggesting that depression severity was associated with reduced survival after adjustment for available demographic, socioeconomic, and clinical factors. The early separation of survival curves further supported the presence of unadjusted survival differences between participants with and without depressive symptoms.

## 4. Discussion

This study suggests that income may partially account for nearly one‐quarter (23.8%) of the observed association between depressive symptoms and mortality in asthma patients. It also highlights a substantial survival disparity: depressed individuals experienced a 9.9‐month reduction in RMST compared to their nondepressed counterparts. This RMST difference represents a restricted survival difference over the predefined 15‐year follow‐up window rather than a lifetime life‐expectancy difference. However, these findings should be interpreted cautiously because the effect size for the continuous PHQ‐9 score was modest in the fully adjusted model, and the association for severe depressive symptoms was attenuated and no longer statistically significant after full adjustment. These results suggest that measured covariates may explain part of the observed association and that residual confounding or limited statistical power in the severe symptom subgroup may remain. Age differences between depression groups may also have influenced the observed mortality patterns because age is strongly related to all‐cause mortality. Although age was included as an adjustment variable in the multivariable Cox models, residual age‐related confounding cannot be fully excluded. The higher crude mortality proportion among participants with depressive symptoms should be interpreted within the asthma‐specific context. This pattern is consistent with broader evidence linking depression to increased all‐cause mortality in general populations [17], as well as prior evidence among adults with asthma [18]. However, direct comparison of absolute mortality proportions across studies should be made cautiously because mortality estimates may differ by follow‐up duration, age distribution, asthma severity, comorbidities, depression definition, healthcare access, and socioeconomic context. Overall, these findings suggest that the survival differences among adults with asthma may reflect both biological and socioeconomic factors. By highlighting these potential socioeconomic pathways, this research supports a more integrative interpretation that may complement traditional mechanistic explanations, such as inflammation [19] and airway remodeling [20], by considering both biological and socioeconomic influences on survival.

Our study suggests that income may serve as a potential explanatory socioeconomic factor in the observed relationship between depressive symptoms and mortality among asthma patients. While prevailing biological theories, such as hypothalamic–pituitary–adrenal (HPA) axis dysregulation and neuroimmune crosstalk, have traditionally dominated explanations of depression‐related mortality [21–23], our findings highlight material deprivation as a relevant socioeconomic factor. The data reveal that nearly half (47.98%) of depressed asthma patients live in poverty compared to 23.50% of their nondepressed counterparts, and PIR accounted for nearly one‐quarter (23.8%) of the observed survival gap in the exploratory mediation analysis. At the same time, high‐income status is markedly lower among depressed patients, with only 18.68% achieving PIR ≥ 3.50 compared to 42.32% in nondepressed individuals.

These disparities mirror established biological risks—such as severe eosinophilic inflammation [24]—yet they differ crucially in their potential for change. Unlike genetic or biological mechanisms that often require targeted pharmacological interventions [25], the income‐related survival gap may be potentially addressable through socioeconomic policies and social reforms. For example, interventions that enhance access to affordable asthma care, expand employment opportunities, and strengthen social safety nets may help reduce this mortality burden [26].

Moreover, the 9.9‐month reduction in RMST among depressed patients further contextualizes the magnitude of this burden. This estimate represents a restricted survival difference over the predefined 15‐year follow‐up window rather than a lifetime life‐expectancy difference. This survival difference is comparable to that observed in patients with poorly controlled persistent asthma [27], yet it raises the possibility that improving socioeconomic conditions may contribute to better survival outcomes, highlighting the value of integrated strategies that address both mental health and social determinants of health. Because the RMST analysis was unadjusted, it should be interpreted as a support absolute measure of survival disparity and considered together with the multivariable Cox regression results. Depression was defined using the PHQ‐9, which assesses depressive symptoms over the preceding 2 weeks and does not provide a clinical diagnosis. Therefore, PHQ‐9 ≥ 10 should be interpreted as moderate‐to‐severe depressive symptoms rather than major depressive disorder. In addition, PHQ‐9 does not distinguish depressive disorder subtypes, and treating depression as a homogeneous construct may have introduced misclassification and limited clinical interpretation. The observed association between depressive symptoms and survival should be interpreted cautiously because important asthma‐specific clinical factors were not fully captured in the NHANES. Psychological factors may interact with asthma pathophysiology and clinical course through disease severity, inflammatory phenotype, healthcare access, and treatment adherence. A recent study also suggested that depressive symptoms may be linked to asthma severity through biological pathways [28]. Cardiovascular comorbidities may also contribute to the observed association as prior meta‐analytic evidence has linked asthma with increased cardiovascular morbidity and mortality [29]. Although our models adjusted for several cardiometabolic factors, including diabetes, BMI, and hypertension awareness, detailed asthma severity indicators, and cardiovascular disease history were not consistently available across NHANES cycles. Therefore, residual confounding by unmeasured asthma severity, inflammatory phenotype, healthcare access, and treatment‐related factors cannot be excluded.

Methodologically, this work addresses a critical gap in observational research. Earlier studies often treated income as a confounder without examining its potential explanatory role [18, 30], which may have limited the understanding of socioeconomic pathways related to survival disparities. Our exploratory mediation analysis extends prior work by evaluating whether income may partially account for the observed association between depressive symptoms and mortality in asthma patients. However, because depression and income were measured at baseline and mediation analysis requires assumptions regarding temporal ordering and no unmeasured confounding, the estimated mediation proportion should be interpreted as exploratory and assumption‐dependent rather than as definitive causal evidence.

The mechanisms underlying the observed association involving income may involve both direct and indirect pathways. Depressive symptoms may be associated with decreased employment and productivity, greater financial strain, and reduced ability to afford essential asthma treatments [31]. At the same time, low income may limit access to healthcare resources and may be associated with delayed treatment, suboptimal disease management, and worse long‐term outcomes [32]. By examining income as a potential explanatory factor, our findings suggest that interventions targeting economic stability—such as financial assistance programs, insurance expansions, and community‐based resource support [33]—may help reduce survival disparities associated with depressive symptoms among asthma patients.

## 5. Limitations

Several limitations should be considered. First, although mortality follow‐up was longitudinal through linkage to the NDI, depressive symptoms and income were measured at baseline in the NHANES. Therefore, the temporal ordering and potential bidirectional relationship between depressive symptoms and income could not be fully determined, limiting the causal interpretation of the mediation analysis. Accordingly, income should be interpreted as a potential explanatory factor within a hypothesized pathway rather than as a confirmed unidirectional mediator. Second, self‐reported data may be subject to recall and social desirability biases. Depression was defined using PHQ‐9 ≥ 10, which reflects moderate‐to‐severe depressive symptoms rather than a clinical diagnosis of major depressive disorder. Potential misclassification should be acknowledged. Asthma status was also self‐reported, and the absence of severity measures represents an important limitation. Third, as the study is based on U.S. NHANES data, the results may not be extrapolated to populations in other countries with different healthcare systems or cultural backgrounds. Because PIR is defined relative to the U.S. federal poverty threshold, low‐ and high‐income categories in this study may not be directly comparable with income categories in low‐ and middle‐income countries. Therefore, the generalizability of these findings should be interpreted cautiously and may be most relevant to countries or settings with similar socioeconomic and healthcare structures. In addition, because detailed geographic identifiers are not available in publicly released NHANES data, we were unable to evaluate regional variation or present geographic mapping of the income distribution within the sampled population. Additionally, missing data may still introduce bias, although the missing covariate information was handled using dummy variables. Finally, although several confounders were controlled for, residual confounding may remain. Detailed asthma‐specific clinical information, including asthma severity, inflammatory phenotype, healthcare access, treatment adherence, asthma control, exacerbation history, and medication use, was not consistently available across NHANES cycles. Detailed contextual variables related to healthcare utilization, such as emergency visits, hospitalizations, and regular access to care, as well as indicators of economic burden, including work productivity, absenteeism, and family‐level asthma burden, were also not consistently available across all included survey cycles and therefore could not be incorporated into the main analysis. These factors may influence both depressive symptoms and survival and may therefore affect the observed association. In addition, the mediation analysis relied on assumptions of no unmeasured confounding, and behavioral or clinical factors may act as confounders or lie on the pathway linking depression, income, and survival. Therefore, the estimated mediation proportion should be interpreted as exploratory and assumption‐dependent.

Future studies should focus on longitudinal data to better clarify temporal relationships and include more diverse populations to enhance generalizability. Investigating the role of genetic and environmental factors in the depression‐mortality link, as well as refining measures of socioeconomic status, would provide valuable insights. Additionally, research on interventions to reduce the impact of socioeconomic disparities on the mental health and mortality in asthma patients is needed.

## 6. Conclusion

In this NHANES‐based observational study of adults with asthma, depressive symptoms were associated with reduced survival, and income may partially account for this observed association. These findings should be interpreted cautiously because temporal ordering between depressive symptoms and income could not be definitively established, and the mediation analysis was exploratory and assumption‐dependent. Because the PIR is based on the U.S. federal poverty threshold, the applicability of these findings to other countries or healthcare systems may be limited. Further longitudinal studies in diverse populations are needed to confirm these findings and clarify the temporal relationships.

**Nomenclature**
NHANES: National Health and Nutrition Examination Survey
NCHS: National Center for Health Statistics
NDI: National Death Index
PHQ‐9: Patient Health Questionnaire‐9
PHQSCORE: Patient Health Questionnaire score
PIR: Poverty–income ratio
DHHS: Department of Health and Human Services
BMI: Body mass index
HR: Hazard ratio
CI: Confidence interval
RMST: Restricted mean survival time
HPA: Hypothalamic–pituitary–adrenal.

## Author Contributions

Zhiyi Wang was responsible for conceptualization, data analysis, methodology, and manuscript drafting. Xiaojing Teng supervised the study, revised the manuscript, and provided final approval.

## Funding

This study was supported by the Construction Fund of Key Medical Disciplines of Hangzhou (Laboratory Diagnostics, Grant 2025HZZD01).

## Disclosure

Both authors have read and approved the final manuscript. All work was performed manually by the authors.

## Ethics Statement

NHANES was approved by the National Center for Health Statistics (NCHS) Ethics Review Board. All participants provided written informed consent before participation. The present study was based on publicly available, deidentified NHANES data and involved secondary analysis of existing data; therefore, no additional informed consent from participants was required.

## Consent

The authors have nothing to report.

## Conflicts of Interest

The authors declare no conflicts of interest.

## Supporting Information

Additional supporting information can be found online in the Supporting Information section.

## Supporting information


**Supporting Information** Table S1: Adjusted hazard ratios for all‐cause mortality by poverty–income ratio categories in asthma patients. Table S2: Association between depressive symptoms and all‐cause mortality stratified by asthma status.

## Data Availability

The NHANES dataset analyzed in this study is publicly available from the NCHS website (https://www.cdc.gov/nchs/nhanes/). All data used in this study can be accessed and reproduced following NHANES data access policies.
