# Supplementary material for: The Association Between Depression and Survival in Asthma: The Mediating Role of Income
Source: Depress Anxiety. 2026 Sep 19;2026:8153352. doi: 10.1155/da/8153352 (PMC13589242; doi:10.1155/da/8153352)
Supplement: Supplementary file 1 [file DA-2026-8153352-s001.docx]

# Supplementary Table：

| **Supplementary Table 1. Adjusted Hazard Ratios for All-Cause Mortality by Poverty-Income Ratio Categories in Asthma Patients** | | | | | | |
| --- | --- | --- | --- | --- | --- | --- |
|  | No Adjust | | Adjust I | | Adjust II | |
|  | HR (95% CI) | *p*-values | HR (95% CI) | *p*-values | HR (95% CI) | *p*-values |
| PIR | 0.78 (0.73, 0.83) | <0.0001 | 0.75 (0.69, 0.81) | <0.0001 | 0.80 (0.74, 0.87) | <0.0001 |
| PIR categorical |  |  |  |  |  |  |
| ≥3.50 | Ref |  | Ref |  | Ref |  |
| 1.30-3.49 | 2.42 (1.79, 3.25) | <0.0001 | 1.98 (1.46, 2.68) | <0.0001 | 1.72 (1.26, 2.35) | 0.0007 |
| <1.30 | 2.76 (2.06, 3.71) | <0.0001 | 3.00 (2.22, 4.05) | <0.0001 | 2.23 (1.60, 3.11) | <0.0001 |
| *p* for trend | <0.0001 | | <0.0001 | | <0.0001 | |
| **NOTE:** Cox proportional hazards regression models were used to examine the association between PIR and all-cause mortality. Results are presented as hazard ratios (HRs) with 95% confidence intervals (CIs) and corresponding *P*-values. PIR was analyzed as a continuous variable and as a categorical variable (PIR ≥3.50 as the reference group). Adjust I model adjusted for Gender, Age, Race/Ethnicity. Adjust II model additionally adjusted for Education Level, Smoking, Diabetes, Marital Status, BMI, Hypertension Awareness. PIR was not included as a covariate in Adjust II because it was the main independent variable. The time variable in the Cox models was PERMTH_INT (time to event in months). Statistical significance was defined as *p* < 0.05. | | | | | | |

| **Supplementary Table 2. Association Between Depressive Symptoms and All-Cause Mortality Stratified by Asthma Status** | | | | | | |
| --- | --- | --- | --- | --- | --- | --- |
|  | No Adjust | | Adjust I | | Adjust II | |
|  | HR (95% CI) | *p*-values | HR (95% CI) | *p*-values | HR (95% CI) | *p*-values |
| **Asthma patients** | | | | | | |
| PHQ-9 <10 | Ref |  | Ref |  | Ref |  |
| PHQ-9 ≥10 | 1.34 (1.04, 1.72) | 0.0242 | 1.92 (1.48, 2.48) | <0.0001 | 1.24 (0.95, 1.63) | 0.1138 |
| **Non-asthmatic participants** | | | | | | |
| PHQ-9 <10 | Ref |  | Ref |  | Ref |  |
| PHQ-9 ≥10 | 1.14 (0.99, 1.31) | 0.0635 | 1.82 (1.58, 2.10) | <0.0001 | 1.34 (1.16, 1.55) | <0.0001 |
| *p* for interaction | 0.2532 | | 0.6070 | | 0.7033 | |

**NOTE:** Cox proportional hazards regression models were used to examine the association between depressive symptoms and all-cause mortality stratified by asthma status. Results are presented as hazard ratios (HRs) with 95% confidence intervals (CIs) and corresponding *P*-values. Depressive symptoms were defined as PHQ-9 ≥10, with PHQ-9 <10 as the reference group. *P* for interaction was calculated by including an interaction term between depressive symptoms and asthma status in the Cox model. Adjust I model adjusted for Gender, Age, Race/Ethnicity. Adjust II model additionally adjusted for Education Level, Smoking, Diabetes, PIR, Marital Status, BMI, Hypertension Awareness. The time variable in the Cox models was PERMTH_INT (time to event in months). Statistical significance was defined as *p* < 0.05.
